# A Descriptive Study on the Impacts of Hyperbaric Oxygen Therapy on Autistic Individuals Using Parent Testimonies

**DOI:** 10.7759/cureus.55648

**Published:** 2024-03-06

**Authors:** Tami Peterson, Tiffany Hosey, Jeffrey Mosteller, Robert Sherwin, Frederick Strale,

**Affiliations:** 1 Hyperbaric Oxygen Therapy, The Oxford Center, Brighton, USA; 2 Hyperbaric Oxygen Therapy, The Oxford Center, Troy, USA; 3 Hyperbaric Oxygen Therapy, Wayne State University School of Medicine, Detroit, USA; 4 Biostatistics, The Oxford Center, Brighton, USA

**Keywords:** : autism spectrum disorder (asd), hyperbaric oxygen therapy (hbot), interrater reliability, parent testimonies, symptom assessment

## Abstract

Introduction

Hyperbaric oxygen therapy (HBOT) has been influential in treating many physical and psychological ailments, including the symptoms of autism. This current study aims to evaluate HBOT parents’ goals and exit interviews describing the positive, negative, or no impacts experienced from the HBOT dives, asking the question, “Are your child’s symptoms improving?”

Methods

Between January 2020 and July 2023, a Class B monoplace hyperbaric chamber (Sechrist 3300H, Sechrist Industries, Inc., Anaheim, California, United States) was used to administer HBOT sessions to patients with autism. Medical-grade oxygen was pressurized to 1.5-2.0 atmospheres absolute at a rate of 1-2 psi/min, with an average oxygen percentage of 100%, for up to five sessions per week. Retrospective descriptive data and patient information through parent testimonials were collected through a chart review of 30 children and one adult with autism who experienced HBOT sessions. Data were presented through exit interviews describing how parents felt about their child’s progress toward goals. Four raters rated parent testimonies on a 5-point Likert scale (1 = Much worse, 2 = Somewhat worse, 3 = Stayed the same, 4 = Somewhat improved, and 5 = Much improved), and an inter-rater reliability estimate using interclass correlation (2) (r = 0.831) was derived, indicating excellent agreement between raters.

Results

Parents/caregivers provided testimony in an exit interview with a registered nurse after the individual with autism received an entire course of HBOT dives. Descriptive statistics resulted in Rater #1 (M = 4.19, median = 4, SD = 0.654): 87.1% of Rater #1 ratings were Somewhat improved and Much improved; Rater #2 (M = 4.23, median = 4, SD = 0.717): 83.9% of Rater #2 ratings were Somewhat improved and Much improved; Rater #3 (M = 4.23, median = 4, SD = 0.560): 93.5% of Rater #3 ratings were Somewhat improved and Much improved; and Rater #4 (M = 4.26, median = 4, SD = 0.631): 90.3% of Rater #4 ratings were Somewhat improved and Much improved. One-way ANOVA resulted in F (3,123) = 0.052, p = 0.984, which indicated a nonstatistically significant mean difference between rater groups.

Conclusions

The current study assessed HBOT parents’/caregivers’ goals and exit interviews, describing the effects experienced from the complete course of HBOT dives on their children/individuals. A majority of parents/caregivers declared that their condition had “Much improved” or “Somewhat improved,” based on the 5-point Likert scale. Based on parents’/caregivers’ testimonies, HBOT was demonstrated as a safe and effective intervention, and side effects were primarily mild and did not lead to treatment discontinuation. As a result of this analysis, we recommend continued use of HBOT for treatment.

## Introduction

Hyperbaric oxygen therapy (HBOT) uses pure oxygen at a higher-than-normal atmospheric pressure, which changes blood (hyperoxemia) and tissue (hyperoxia) oxygen levels. Supersaturating the body with high doses of oxygen induces antimicrobial, immunomodulatory, and angiogenic effects [1]. Results on treatment outcomes with HBOT as a method of symptom reduction in the treatment of children with autism spectrum disorder (ASD) vary [2-9].

In a prospective pilot study of autistic children, HBOT at a maximum pressure of 1.5 atmospheres absolute (ATA) with up to 100% oxygen was found to be safe and well tolerated. HBOT did not worsen oxidative stress and significantly decreased inflammation, as measured by CRP levels. Parental observations support improvement in several domains of autism [2]. In a double-blind, randomized controlled study, children with autism who received HBOT at 1.3 atm and 24% oxygen for 40 hourly sessions significantly improved overall functioning, receptive language, social interaction, eye contact, and sensory/cognitive awareness compared to children who received slightly pressurized room air [3].

In another study, improvements were found in younger children under study post-therapy for sociability, sensory/cognitive awareness, health/physical/behavior, and sound-sensitive items. In older children, improvements were noted for health/physical/behavior, emotional response, adaptation to change, and total score [5].

There have been multiple literature and systematic reviews concerning the use of HBOT in treating autism, which have reported mixed results ranging from no benefit to some reporting promising results [6-8]. Lasheen et al. concluded that HBOT improved auditory attention and memory in cases of autism and recommended increasing the sample size in further studies on the effect of HBOT in cases of autism with more HBOT sessions [9].

Meyer noted that numerous studies have demonstrated that HBOT is an effective treatment for children with autism [10]. The pressures utilized during treatment with HBOT (1.5 atm/100% oxygen maximum) are proven to improve the common physiological abnormalities in individuals with ASD, such as cerebral hypoperfusion, oxidative stress, inflammation, and mitochondrial dysfunction. Furthermore, studies that targeted the behavioral measurements in ASD also showed positive results, even though most of those studies did not use control groups. The two studies that did utilize control groups, however, had opposing outcomes. Based on the studies performed, overall, the use of HBOT appears to be a promising treatment for children with ASD.

El-baz et al. reported significant improvement in the Autism Evaluation Treatment Checklist (AETC) scale in total score, subscales (sociability, sensory/cognitive awareness, health/physical/behavior, and communication), and the Childhood Autism Rating Scale (CARS) after treatment with at least 20 sessions of HBOT at 1.5 ATA and 100% oxygen [11]. The average improvement of the AETC total score in all children was 32.1%, and the average CARS score was 15.1%. Rossignol and Rossignol [12] noted that six children completed 40, one-hour sessions of low-pressure HBOT at 1.3 ATA and 28-30% oxygen and reported an average improvement of the AETC total score in all children of 22.1% and an average improvement of the CARS score in all children of 12.1%.

This current descriptive study aims to evaluate HBOT regarding the goals of the parents/caregivers of children with autism and exit interviews describing the impacts on the children experienced from the HBOT dives. No studies to date have examined parent/caregiver testimonies relative to the impacts of HBOT after 40 dives on their respective children/individuals.

## Materials and methods

Between January 2020 and July 2023, a Class B monoplace hyperbaric chamber (Sechrist 3300H, Sechrist Industries, Inc., Anaheim, California, United States) was used to administer HBOT sessions to patients with autism at The Oxford Center, Brighton, United States. Medical-grade oxygen was pressurized to 1.5-2.0 ATA at a rate of 1-2 psi/min, with an average oxygen percentage of 100%, for up to five sessions per week. Patients were monitored for adverse events by trained hyperbaric technicians. The hyperbaric chamber was depressurized from 1 to 2 psi/min back to 1.0 ATA.

Before entering the hyperbaric chamber, each parent/caregiver was given a pre-treatment screening (consented) by the Certified Hyperbaric Technician (CHT), including goal setting and a review of their child/individual’s medical history and what to expect in terms of benefits/risks. The children/individuals were also instructed to equalize the pressure in their ears, like being a passenger on a commercial airliner, which may cause discomfort during the hyperbaric process.

Patients were provided hospital scrubs to wear during the hyperbaric session. They were instructed to remove any metal, i.e., jewelry, glasses, dentures, contact lenses, and other items that may undergo damage due to oxygen hyper-pressurization. Each patient was administered medical-grade pure oxygen in the chamber, lying down. The chamber was sealed and gradually pressurized by the CHT, who communicated with them through the intercom. Patients were treated for between 30 minutes and two hours. After the treatment, the chamber was slowly depressurized, and the patient was released. They were then advised to drink fluids and rest before returning to normal activities.

Parents/caregivers gave feedback, including exit interviews after experiencing the HBOT sessions, describing how their child/individual physically felt and any progress made toward goals. Data was obtained via retrospective chart review from the AdvancedMD database to gather demographic information, discovery session goals, and exit interview testimony. The parent/caregiver testimonies were entered into a Microsoft Excel spreadsheet (Microsoft Corporation, Redmond, Washington, United States), and the discovery goals and exit interviews were visually analyzed and interpreted.

The authors constructed a 5-point Likert scale with 1 = Much worse, 2 = Somewhat worse, 3 = Stayed the same, 4 = Somewhat improved, and 5 = Much improved, based on the exit interview testimonies on discovery goals versus the current condition after 40 dives. Four raters then decided which 5-point Likert ranked category was deemed most appropriate, based on the content of the patient testimonies. The four raters were “independent raters” and were not directly involved in collecting the parent testimonies.

Statistical methods

IBM SPSS Statistics for Windows, Version 29.0.0 (Released 2022; IBM Corp., Armonk, New York, United States) [13] was used for all descriptive analyses. Demographics were summarized for all subjects. Summary statistics (e.g., number of subjects, mean, standard deviation, median, minimum, and maximum) were generated for all continuous variables (i.e., age, HBOT treatment months, dives, and Rater 1-4). Frequencies were reported for gender and rater ratings. Nominal alpha (α) of 0.05 was specified, and statistical significance was declared for p < 0.05. All statistical results were reported via text and table presentation. No conventional power analysis was conducted, as would be the case with studies involving quantitative effect sizes.

Inter-rater reliability

A two-way random effects model was computed where people’s effects and measures effects are also random. We used interclass correlation (ICC) (2), which is used when multiple measurements are taken from each averaged rater. The ICC (2) value was 0.831 (95% CI: 0.707-0.911), indicating excellent agreement between the raters [14-16]. This value was greater than the average Pearson r (0.546), suggesting that the ICC (2) was more sensitive to the variability among raters and measurements. Cronbach’s alpha for the four raters was r = 0.827.

## Results

Descriptive statistics

For the sample of 31 children, regarding age (M = 6.58, SD = 4.85), the median was 5, the minimum was 2, and the maximum was 26. There were 24 males (77.4%) and seven females (22.6%). Regarding dives (M = 44.61, SD = 12.62), the median was 40, and the mode was 40. The minimum was 38, and the maximum was 86. With months of treatment (M = 2.31, SD = 1.35), the median was two months, the minimum was one month, and the maximum was seven months. For dives (M = 44.61, SD = 12.62), the median was 40, the minimum was 38, and the maximum was 86. For months of HBOT treatments (M = 2.31, SD = 1.35), the median was 2, the minimum was 1, and the maximum was 7. There were no missing values. Table 1 below reports rater ratings and parent testimonies.

**Table 1:** Rater ratings and parent testimonies 1 = Much worse; 2 = Somewhat worse; 3 = Stayed the same; 4 = Somewhat improved; 5 = Much improved ABA, applied behavior analysis; ASD, autism spectrum disorder; BCBA, Board Certified Behavior Analyst; BM, body movement; CBD, cannabidiol; HBOT, hyperbaric oxygen therapy; NT, nuchal translucency; PANS, pediatric acute-onset neuropsychiatric syndrome; ST, speech therapy; TOC, The Oxford Center

Patient number	Age	Rater #1	Rater #2	Rater #3	Rater #4	Discovery session goal	Parent testimony after completion of 40 HBOT dives
1	7	4	4	4	5	Improve speaking, increase calmness, and improve socialization	Heard “random words.” Learning new words: said “baby”. Still “pretty hyper.” No improvement in socialization. OT pushed him a lot more, and he made a lot of progress. Made more progress at TOC than in previous therapies. OT started with gross motor and then went to find motor. Mom is very happy with his progress. Mom said, “Speech is most improved.” More babbling.
2	5	4	4	4	4	Be asking mom for things, paying more attention, and communicating more	First two weeks: less hyperactive. Followed instructions. The last two weeks feel like regressed. Since in the US there is no gluten-free, dairy-free, or soy-free diet, feels like this affected progress. Currently sleeping better. Now taking naps consistently. Now looks closely at Mom’s lips, trying to understand what Mom is saying. When at home, has meltdowns two to three times per week. While at TOC for a month, has only had two meltdowns. Adjusted very well to being in the US.
3	5	3	4	4	3	More speech	More detoxing, more BMS. Tough time with the pandemic. Behavior is increasing due to the absence of ABA. He is doing well in ABA now and has just started back. He used to be a zombie, but now he reacts like a normal kid. Sleep: CBD oil stopped six weeks ago. Starting again on Sunday.
4	5	4	3	4	4	Hear him talk again and potty train	Said a few words since starting HBOT: “Mama” and “Up.” Disappointed that Dr. Bogner can’t read the results of the NT/genetic test. Good BMs: Sleeping better. More aggressive the last few days.
5	6	5	5	5	5	Write letters correctly and brush his teeth on his own	Happier. Thankful for the opportunity. Able to handle a lot of situations better. Saw improvement. More expressive language. Sang Jesus Loves the Little Children with Dad. More clear speech. Said bye-bye unprompted and waved. Calmer and can sit and watch TV without running. More social: wants to be with parents and wants to be part of kids playing. Little more interest in toys again. More eye contact. Big improvement in receptive language delay. Responds when first time asked. Good at bike riding. Little less impulsive. Stops when told, moves over, looks back, and waits for family.
6	16	4	4	4	4	Help him understand how to help himself. Time management with studies. Getting off ADD Rx. Showing anger and frustration lately. Looking to the future when leaves home. Says “I don't know” a lot	Noticed a difference in communication. Still gives one-word answers but has more conversations and expresses himself. Was able to calmly explain why he disagreed with Dad and explain what he wanted to see. Still have quiet moments. Still doesn’t recognize what he needs or doesn’t want to change. Not able to recognize changes from treatment. Time management, we will see. Has gotten better at putting technology away when asked. (Exit interview completed after 35 dives)
7	3	4	4	4	5	More verbal. More communication and eye contact. Diversity between offers, not just responding to dad	Way more eye contact. Caught him singing/sounds of his favorite songs. Now imitating. If you bring a device, he will hold it to your face. Won’t say much but will communicate his frustration now. More awareness.
8	6	4	4	4	4	Picking up different items and knowing what they are consistently points to full intentionality	Grandma said he did improve some. He sat and played for a period of time. Better at following directions.
9	4	4	4	5	4	Speech, communication, and expressing emotions/needs. Prepare for school. More focus on “sitting in school longer”	School is going very well. Took time to adjust. Was screaming a lot, but has decreased. Increased eye contact, more variety of pretend play, and repeating back more words.
10	3	5	5	5	4	More communication. Currently one-word repeats. Will not request. Won’t interact with other kids – social	More communicating. Requesting. Anything off the wall. Better eye contact. When took to the park, wanting to play with other kids. She’s friendlier. Now paying more attention to others.
11	16	3	3	3	3	More language. Decrease tics. Settle down from PANS. Doing a lot more talking. Express how she is feeling emotionally and physically. Improve reading	Mom feels symptoms get worse at times of menstrual period. Decreased focus. Increased tics. Cyclical flair-up of the above symptoms. Mom doesn’t feel HBOT has improved symptoms. First night after HBOT, much better focus. Maybe small improvement in vocalization.
12	3	5	4	5	5	Improved verbalization. Doing things that are age appropriate	Doing super. Lots more eye contact, more social. Sleep is better. Praying with family. Eating is atrocius. Waiting on genetics. More words. The words are there just trapped. Action figures and dinosaurs and he’s playing with them and babbling. Plays with dinosaurs on the right mat. Notices when the wrong mat is used. Played with cars and trains for 15 minutes on his own. Loves the water play. Far more focused on water play now. Doing well with focus and attention. Going to the potty here hasn’t gone home yet. Started liking books here, so will use the potty at home.
13	4	3	3	3	3	To be able to go places with the family. Go to a birthday party; couldn’t control screaming. Brought too much attention. Public places are hard	Screaming is still there. Sleeping is better through the night without disturbing everyone else. Out of HBOT can only benefit from there. Has decreased depressive mood. Still has screaming if don’t want to be there. BCBA says more verbally. She doesn’t think functionally will use words to get what she wants.
14	9	4	5	5	5	I want him to heal, be seizure-free, and take care of himself	More energy. Constipation is a little better. Seizures are better at night. Don’t see/feel them anymore. Sleeps better now. The ABA therapist says he’s improving in general. More communication. The speech pathologist hears more sounds.
15	9	3	4	4	4	Speech	Speech is about the same. He understands and listens more to his father. His speech is still mostly vocalizations. He desperately wants to communicate and sometimes cries when he cannot. From tech’s perspective, he is babbling less and actively trying to communicate through vocalizations.
16	4	5	5	4	5	Hear him talk again. Increase in communication	Noticed a tremendous difference. Can take to a store and out for a walk without being afraid he will run away. Attention to detail. So much better eye contact and understanding of emotions. Trying new food. Toilet training is much better. Making many more sounds and vocalizations.
17	8	4	4	4	5	Short attention span in school and at home. Coordination, global delays in speech, fine motor, and gross motor skills	Made a lot of improvement/growth. More empathy, new skills, better social skills. Since HBOT, didn’t see a major change. Better at wiping self, better emotional regulation, and haven’t seen more interaction with their brother. Asking mom to play more. Very snuggly: has been jealous of new siblings. Back to being more affectionate. Love/hate relationship with brothers.
18	5	4	5	4	4	No symptoms of autism, more engaged, and easier communication. Better social interaction	Better social interaction. Still has signs of autism. Not sure about communication. More interaction with peers since ABA. Hoping to start school in the fall. ASD symptoms improved: will interact with people longer. Will interact when friends come over. Potty trained now: last six months with reminders. Met a lot of his goals through ABA. Very happy they went with the program.
19	3	4	5	5	5	Improvement in speech	Going well. More babbling sounds. Attention span longer: five minutes started to 45 minutes. Better able to follow directions and simple commands.
20	6	5	5	4	4	Able to focus on learning and reading. Ease the transition to first grade. Improve comprehension	Focus improved. Teachers at school noticed too. Better conversation. Improved emotional intelligence. Asking appropriate questions. Positive behaviors are noticed by teachers. Gluten-free diet since the start of HBOT.
21	6	4	3	4	4	Get him speaking. Nutrition: Mom thinks contributing to sensory issues	Hard to tell if improvements are from HBOT/therapies or if they’re from time. Sleeping better. Used to wake up in the middle of the night and do gymnastics. Hasn’t happened in quite a while. Speech: babbles and hasn’t put it all together yet.
22	5	5	5	4	5	Speak a sentence and tell his name. Keep up with the other kids	Loves the friendly faces. He loves HBOT. Vocalizing more. His ABA therapist noticed better focus. Able to verbalize and ask for what he wants. Words are clearer. Focus improved. Has helped self-esteem.
23	5	5	5	5	4	Decrease stimming. Help with inflammation, other issues resolved, more prepared for school, and going to public school	Been good, seen improvements, a lot more calm, and a lot less stimming. Sat by self for a haircut. Cedar point: drank out of the cup by self. Teachers at ABA noticed he asked a lot more questions and was calmer. Helped more than they thought. Family noticed he listened more and a lot more calmly.
24	8	4	5	4	4	One-word answers to questions. Decrease hyperactivity. Improvement in speech	Getting out multisyllable words. Will say two to three words at a time if prompted now. Lost six teeth during HBOT; getting four new teeth. Less hyperactivity. Took training wheels off her bike.
25	3	5	4	4	4	Speaking, improvements in speech, two-word phrases not prompted, "Help me." Get needs met. Not fall further behind other kids in ST	July 21: Cousin came. He ran up to greet her, touched her shoulder, looked her in the eye, and said “Hi.” Huge thing. July 25: At church, he made the sign of the cross. Vocalizations: prompted “a book,” “a ball,” said his friend’s name, and said “Papa.” Last weekend actively played hide and go seek, taking turns, counting, and hiding. Momentous to hear. All he had said before was “help,” “bye,” and “set go.” Saying “one, two, three, abo” (blast off) with Buzz Lightyear motions.
26	26	5	5	5	5	Conversational speaking. To be able to pick up the phone to say "Mom, where are you? I’m lost. Can you help me?"	He’s been more expressive. Less fatigue. More focus. More impromptu. Advocating for himself. Hearing “Hi.” Starting to care. His employer has noticed a change: more conversational. Pleased with what we are seeing. Telling mom about his day. Feeling better; more interested. Spelling better. Wears a mask all day at work.
27	5	4	5	4	5	Potty training and communication	Potty training is still ongoing. Sleeps better, listens better, is more vocal, is more aware of his body, and uses hands more. New noises and comprehending more.
28	8	4	4	4	4	Impulsivity and hyperactivity	She is still hyperactive and impulsive. Speech, language, and attentiveness have improved a lot. Uses more words. She is still aggressive.
29	5	4	4	5	4	Hear some words. Better focus	Better focus. Listens better. Interacts better with people. Less impulsiveness, more engagement, and starting to say echoic speech.
30	4	4	4	4	4	Decrease aggression. Improve communication/verbal/sign. Improve sleep	Improved a lot. Not as aggressive. Biting stopped. Flapping, but not as much. Sleep improved. Speech has not improved; “Dada” before, but now mom thinks regressed. Maybe being stubborn. Response time got better.
31	2	5	4	4	4	Verbalize. Better focus. Better eye contact. More engaged with parents	Sleeping better. Mom feels better focused. Better eye contact. Better recognition of people. Not speaking yet; making soft noises with music and reading stories. Feels he is more engaged with them. Eating better.

The ratings from each rater are summarized as follows: Rater #1 (M = 4.19, SD = 0.654, median = 4): Rater #1 rated zero patients (0.0%) as Much worse, zero patients (0.0%) as Somewhat worse, four patients (12.9%) as Stayed the same, 17 patients (54.8%) as Somewhat improved, and 10 patients (32.3%) as Much improved; Rater #2 (M = 4.23, SD = 0.717, median = 4): Rater #2 rated zero patients (0.0%) as much worse, zero patients (0.0%) as Somewhat worse, five patients (16.1%) as Stayed the same, 14 patients (45.2%) as Somewhat improved, and 12 patients (38.7%) as Much improved; Rater #3 (M = 4.23, SD = 0.560, median = 4): Rater #3 rated zero patients (0.0%) as Much worse, zero patients (0.0%) as Somewhat worse, two patients (6.5%) as Stayed the same, 20 patients (64.5%) as Somewhat improved, and nine patients (29.0%) as Much improved; and Rater #4 (M = 4.26, SD = 0.631, median = 4): Rater #4 rated zero patients (0.0%) as Much worse, zero patients (0.0%) as Somewhat worse, three patients (9.7%) as Stayed the same, 17 patients (54.8%) as Somewhat improved, and 11 patients (35.5%) as Much improved.

Table 2 below reports rater means, standard deviations, and medians.

**Table 2:** Descriptive statistics for raters Results are reported as n, mean, standard deviation, and median.

Descriptive statistic	Rater #1	Rater #2	Rater #3	Rater #4
n	31	31	31	31
Mean	4.19	4.23	4.23	4.26
Median	4	4	4	4
Standard deviation	0.654	0.717	0.56	0.631

In order to determine the equality of rater means, a one-way ANOVA was conducted, F (3,123) = 0.052, p = 0.984, indicating a nonstatistically significant difference (p > 0.05) between rater means. See Table 3 below.

**Table 3:** One-way ANOVA on rater means The data has been represented by the source of variation, the sum of squares, degrees of freedom (df), mean square, F, and p-value.

Source of variation	Sum of squares	df	Mean square	F	p-value
Between groups	0.065	3	0.022	0.052	0.984
Within groups	49.613	120	0.413		
Total	49.677	123			

## Discussion

This study found that 87.1% of Rater #1 ratings were Somewhat improved and Much improved, 83.9% of Rater #2 ratings were Somewhat improved and Much improved, 93.5% of Rater #3 ratings were Somewhat improved and Much improved, and 90.3% of Rater #4 ratings were Somewhat improved and Much improved.

This small study may be a positive step in showing the link to multiple improvements for individuals with autism resulting from HBOT. While preliminary and descriptive, these data are consistent with the potential benefits of several known medical pathologies common in autistic patients, including reducing inflammation and enhancing host response to gut bacterial colonization. Specifically, three known processes of hyperbaric oxygenation seem to have contributed to these results.

HBOT contributory processes

Hyperbaric Oxygenation of Blood and Tissues

Increasing the pressure and percentage of inspired oxygen enhances the partial pressure of oxygen (PaO2), measuring arterial oxygen pressure and indicating efficient lung-to-blood oxygen transfer. It reflects how well oxygen can move from the lungs to the blood. HBOT treatments result in PaO2 ranging from 100 mmHg to 1,483 mmHg. This process causes an increase in the penetration of oxygen into the tissue to 247 µM from 64 µM. This allows tissues experiencing hypoxia due to inflammation to get the needed oxygen [17].

Vasoconstriction

One of the brain’s automatic responses to hyperbaric oxygenation is constricting the arteries to reduce blood flow and oxygenation. Reducing blood flow reduces the fluid flowing into edematous tissue and relieves the compartment pressures, allowing stressed and stretched capillaries to relax and open, restoring normal blood flow [18].

Host Response to Bacterial Infection

It is well known that hyperbaric oxygenation helps kill bacteria by oxidative burst, enhancing the process of phagocytosis, which requires oxygen to occur and slows in hypoxic tissue, and synergism with certain antibiotics in enhancing bacterial clearance [19,20].

Oxygen is required for correct bodily function, and this naturally available energy source is used in cellular respiration, metabolism, adenosine triphosphate production, detoxification, immune system support, brain function, and the healing of tissue and bone. Hyperbaric chambers create an environment where the administration of a concentrated form of oxygen is arguably the most vital source of life. Despite the consistent research affirming its safety, the broader mainstream medical community is hesitant to accept the application for conditions such as ASDs [20].

Oxygen quality

For this study, we needed to prove the quality of our treatment gas. We were able to accomplish this with the help of our cryogenic oxygen provider. Liquid oxygen is rigidly controlled and must meet established quality standards. The standards include an oxygen percentage of ≥99.5%, a water vapor content of ≤6 ppm, and no detected odor. Each lot of gas has the results of testing the lot along with an assigned lot number. The vendor provides a copy of their standards compliance. Through this, a running record of each lot at each of our locations is kept. We filed the results, and with an ongoing spreadsheet, we calculated the current average oxygen percentage for the time period of the study. Through this, we were able to, with a strong certainty, know that for the study period, our average oxygen percentage was 99.803% [21].

The benefit of HBOT for autistic individuals

Remaining open-minded about the benefits of oxygen has proven to be impactful for so many families. Notably, the parent/caregiver feedback in this study demonstrated a pattern of positive impacts and a high safety incidence.

The word “spectrum” in the name of the condition signifies a diverse range of symptoms, characteristics, and challenges. The heterogeneity of the diagnosis demands varied treatments and flexible approaches. Individuals with autism can differ significantly in their cognitive abilities and communication skills, which is why varied responses to treatments make it difficult to establish universal interventions. Tailoring care plans to each person’s unique needs enhances outcomes.

El-Tellawy et al. found positive effects with HBOT combined with Tomatis sound therapy in children with autism [8]. El-baz et al. found that with 20 children with autism, there was a statistically significant increase in the ratio of regional cerebral blood flow (RCBF) to white matter after HBOT in different brain regions when compared to their levels before HBOT [11].

The qualitative information given by this sample of parents/caregivers stands as a first step in establishing a foundation for future prospective investigations. It may provide insights into the experiences of autistic individuals as reported by parents/caregivers. Through exit interviews, researchers can identify variables crucial for consideration in the design of future clinical trials.

Lasheen et al. found that children with autism showed improvement in both auditory attention and auditory memory after HBOT in their experimental group versus their control group [9]. Similar to Rossignol et al., parental/caregiver observations support anecdotal accounts of improvement in several domains of autism [2].

Quantitative evidence was reported by Rossignol et al. [3], stating that children with autism who received HBOT at 1.3 ATA and 24% oxygen for 40 dives had significant improvements in overall functioning, receptive language, social interaction, eye contact, and sensory/cognitive awareness compared to children who received slightly pressurized room air.

Kostiukow and Samborski reported that parental observations support anecdotal accounts of improvement in several domains of autism [6]. Lasheen et al. found that children with autism showed improvement in both auditory attention and auditory memory after HBOT [9]. Meyer reported that, although HBOT is not yet approved by the United States FDA, several studies performed internationally have proven its efficacy in treating people with autism [10].

El-baz et al. [11] found a statistically significant increase in the ratio of RCBF to white matter after HBOT in different brain regions when compared to their levels before HBOT. They emphasized that HBOT is a treatment that has recently become quite popular in the ASD community. Its benefits are across a wide range of autistic traits, as it improves language and increases awareness, behavior, and socialization. We recommend that the child undergo a minimum of 40 sessions. Some children may require up to 80 sessions to see benefits. HBOT can be used for any age, but a better effect is obtained with early intervention and any degree of autism. However, a better effect is obtained in mild and moderate cases. Further research is needed on a large scale in this area as a new modality of treatment [11].

To combat the placebo effect and address criticism from the research community, rigorous, controlled studies with validated outcome measures are essential. While alternative or complementary treatments, like HBOT, should not be promoted as a guaranteed solution, rejecting the potential benefit globally would be a mistake.

The results of this qualitative study support claims that HBOT positively impacted most subjects. However, the authors exercise caution in reporting widespread application to anyone diagnosed with autism. After considering the study’s context and limitations, there is optimism that the data will serve as a framework for subsequent research.

HBOT may have early evidence of inducing an efficacious effect in children and adults to reduce symptoms. In this sample, HBOT side effects were primarily mild and did not lead to treatment discontinuation. Remaining open-minded about the benefits of HBOT has proven impactful for many families. Qualitative studies on the effectiveness of HBOT with autistic children/individuals are essential and much needed for several reasons. Parent/caregiver testimonies can help healthcare providers understand the impact of HBOT on the quality of life of autistic children and tailor treatment plans accordingly. Also, qualitative studies can help identify potential benefits and risks associated with HBOT that may not be evident in quantitative research and identify areas that need further investigation. Descriptive studies play a crucial role in enhancing our understanding of the effectiveness of HBOT in treating autistic children, informing clinical practice, and guiding future research. However, more extensive, well-designed studies are needed to further evaluate the effects and mechanisms of HBOT. Due to this analysis, we recommend continued use of HBOT for treatment.

Limitations

This study is small and is likely not to represent the entire population. Also, it is prudent to be conservative about concluding a compilation of comments. This study does not present hard evidence for a cause-and-effect relationship. The potential for a placebo effect should not be overlooked. Due to the nature of this qualitative study, it was also difficult to fully adjust for potential confounders. The authors evaluated the exit interviews for classification into the various 5-point Likert categories. Many subjects were on one or more treatment therapies and/or medicines throughout their hyperbaric treatment therapy. This was a single-center retrospective, small qualitative study of self-selected patients for HBOT and was not based on personal preferences, opinions, or financial means. However, our research nevertheless has important implications for the effectiveness of HBOT treatments and adds to an ever-growing body of data.

## Conclusions

The current study assessed HBOT parents’/caregivers’ goals and exit interviews, describing the effects experienced from the complete course of HBOT dives on their children/individuals. Most parents/caregivers declared their condition was “Much improved” or “Somewhat improved” based on four separate raters’ ratings on the 5-point Likert scale. Based on parents’/caregivers’ testimonies, HBOT was demonstrated as a safe and effective intervention, and side effects were primarily mild and did not lead to treatment discontinuation. As a result of this analysis, we recommend continued use of HBOT for treatment.
