# Correction: A Descriptive Study on the Impacts of Hyperbaric Oxygen Therapy on Autistic Individuals Using Parent Testimonies

**DOI:** 10.7759/cureus.c247

**Published:** 2025-08-13

**Authors:** Tami Peterson, Tiffany Hosey, Jeffrey Mosteller, Robert Sherwin, Frederick Strale,

**Affiliations:** 1 Hyperbaric Oxygen Therapy, The Oxford Center, Brighton, USA; 2 Hyperbaric Oxygen Therapy, The Oxford Center, Troy, USA; 3 Hyperbaric Oxygen Therapy, Wayne State University School of Medicine, Detroit, USA; 4 Biostatistics, The Oxford Center, Brighton, USA

The Conflicts of Interest statement has been updated to declare the following:

Financial relationships: Tami Peterson, Tiffany Hosey, Jeffrey Mosteller, Robert Sherwin and Frederick Strale Jr. declare employment from The Oxford Center.

